# Preoperative carbohydrate volume optimization in ERAS-guided minimally invasive gastrectomy: a single-blinded RCT assessing gastric residuals and metabolic safety profiles

**DOI:** 10.3389/fsurg.2025.1594956

**Published:** 2025-09-25

**Authors:** Chaojun Ma, Zhiyuan Li, Xin Fan

**Affiliations:** Department of Gastrointestinal Surgery, Affiliated Hospital of Jiangsu University, Zhenjiang, Jiangsu, China

**Keywords:** oral carbohydrate, ERAS, gastric cancer, preoperative drinking, gastric residue volume, perioperative complications

## Abstract

**Background:**

By counting the volume of gastric contents aspirated under gastroscopy after tracheal intubation, ph and monitoring some indicators in the perioperative period. To assess the effect of preoperative oral administration of different doses of carbohydrates on ERAS results in patients undergoing laparoscopic gastric cancer resection.

**Methods:**

The present study was conducted as an investigator-initiated, randomised controlled, parallel group, equivalence trial. The study population comprised 66 patients diagnosed with gastric adenocarcinoma, who were randomly assigned to either group A or group B. Patients in group A consumed 200 ml of 5% dextrose solution 2 h prior to the operation, and patients in group B consumed 400 ml of 5% dextrose solution 2 h prior to the operation. gastric contents were suctioned through a gastroscope immediately after endotracheal intubation. The main observation indexes were preoperative gastric residual volume.

**Results:**

The final study analyzed 60 patients (30 in group A and 30 in group B). The baseline characteristics of the patients in both groups were comparable. There were no significant differences between the two groups in terms of residual stomach volume (36.4 ± 9.6 vs. 37.7 ± 8.8 ml, *P* = 0.565), pH (2.57 ± 0.49 vs. 2.62 ± 0.53, *P* = 0.67), variability in suction volume per aspiration (SVV: 12.69 ± 3.21 vs. 11.85 ± 2.56, *P* = 0.105), and incidence of postoperative complications (13.3% vs. 16.7%, *P* = 0.105). 13.3% vs. 16.7%, *P* = 0.105) Compared with Group A, there was a difference in the degree of discomfort before surgery among patients in Group B. (thirst score: 2.03 ± 1.15 vs. 1.57 ± 1.01, *P* = 0.049; hunger score: 3.1 ± 1.3 vs. 2.3 ± 1.1, *P* = 0.002).

**Conclusion:**

In gastric cancer patients undergoing elective laparoscopic radical gastric cancer surgery, consumption of 200 ml and 400 ml of carbohydrate beverages 2 h before surgery did not significantly increase gastric residual volume, acidity, or perioperative complications, and no significant differences in intraoperative hemodynamics were observed. Increasing preoperative oral intake within safe limits may further reduce thirst and hunger scores. Individualized adjustment of rehydration volume is recommended for elderly or obese patients.

## Introduction

Enhanced Recovery After Surgery (ERAS) promotes patient recovery by optimizing perioperative management. Its core measures include preoperative education, avoidance of bowel preparation, multimodal analgesia, and early transoral feeding (1, 2). Perioperative fluid management, as a key link in the ERAS pathway, directly affects the postoperative recovery outcome. Oral administration of clear liquids (e.g., carbohydrate drinks) 2 h before surgery has been widely recognized as an important measure in ERAS, and the American Society of Anesthesiologists (ASA) guidelines explicitly recommend its use in patients with normal gastrointestinal function (3, 4), and studies have confirmed that this strategy relieves hunger, thirst, and metabolic disorders, shortens hospital stay, and does not increase gastric volume or the risk of malabsorption (5); However, fluid management requires precise balance: Over-hydration may induce intestinal edema and postoperative obstruction, while under-hydration causes organ hypoperfusion—both contrary to the Goal-Directed Fluid Therapy (GDFT) principles advocated by ERAS (6).

Although the 2 h preoperative oral carbohydrate strategy has been well established in colorectal surgery (7), its implementation in gastric cancer surgery is still controversial. While the ERAS Society recommends comparable management for gastrectomy (6), patients with gastric cancer often suffer from gastric dyskinesia and delayed emptying (8), Retained gastric contents can increase surgical difficulty, leading to inconsistent clinical adoption of preoperative carbohydrate loading. Previous studies suggest ERAS protocols—including reduced fasting and preoperative carbohydrates—may benefit gastric cancer patients (9–11, 41) but these primarily assessed safety and subjective experiences. They lacked systematic evaluation of individual differences (e.g., age, BMI, gastric emptying rate) and optimal fluid volumes.

This study innovatively addresses these gaps by: (1) quantifying gastric emptying through direct intraoperative gastroscopy with residual fluid aspiration and pH measurement, and (2) correlating these findings with hemodynamic parameters (e.g., stroke volume variation, cardiac output). These results provide direct evidence for developing individualized rehydration protocols, enabling dose optimization in special populations like the elderly and obese.

## Methods

### Study design

This study was an investigator-initiated randomized controlled, parallel-group, equivocal clinical trial to evaluate the safety and efficacy of preoperative oral carbohydrate solution (5% dextrose) in patients undergoing laparoscopic radical gastric cancer surgery under accelerated rehabilitation surgery (ERAS) pathway. The trial protocol was reviewed and approved by the Research Ethics Committee of Jiangsu University Hospital [gov: ky2024K0102)], and all patients signed a written informed consent before enrollment.

Inclusion criteria include (1) Age 60–80 years; (2) Histologically confirmed gastric adenocarcinoma; (3) American Society of Anesthesiologists (ASA) score I-III. (4) Preoperative cT2-4aN0-2 staging (based on AJCC 8th edition TNM staging criteria) as assessed by gastroscopy, ultrasonography and/or abdominal CT; (5) Suitable to undergo ERAS laparoscopic radical gastric cancer surgery. (6) All of them were the 1st operation of the day and had a smooth surgical procedure. (7) Patients signed an informed consent form to participate in the trial voluntarily.

Exclusion criteria include: (1) preoperative radiotherapy or chemotherapy; (2) history of gastrectomy, history of gastric cancer treatment, or history of major abdominal surgery; (3) pyloric obstruction, impaired intestinal function; (4) history of diffuse peritonitis; (5) preoperative comorbid gastrointestinal disorders (e.g., gastrointestinal dysdynamia, hemorrhage, perforation, obstruction, etc.); (6) the need for emergency surgery or palliative surgery; (7) distant metastasis of the tumor; (8) diabetes or other endocrine hormone abnormalities; (9) severe dysfunction of the heart, lungs, brain, kidneys, or other organs; (10) malaise or impaired circulation of unperipheral blood; (11) difficulties in airway management; (12) direct participation in the trial of the sponsor, the investigator, or a member of his/her family; (13) participation in other clinical trials within the 3 months prior to the trial; (14) contraindications to accelerated surgical rehabilitation or the inability to complete the ERAS procedure; (15) judged by the investigator to be unsuitable for participation in this trial.

After initial screening, patients were randomly assigned in a 1:1 ratio to the 200 ml group (200 ml) and the 400 ml group (400 ml) using a computer-generated random sequence. Allocation concealment was implemented using the sealed envelope method to minimise selection bias. To minimise the influence of time-related confounding factors such as seasonality while ensuring similar progression between groups, 66 patients were evenly divided into 11 groups, with randomisation conducted within each group. A random number generator was used to generate six random numbers for each segment, creating a random code table. Unblinded roles: surgeons and anaesthetists (due to the need to perform dose-specific interventions); blinded roles: endoscopists, laboratory technicians, follow-up evaluators, and statisticians.

### Intervention

The study intervention was the consumption of a carbohydrate beverage containing a 5% glucose solution (China Otsuka Pharmaceutical, Beijing, China). Prior to consumption, all oral solutions were stored at 37°C.The patients consumed the solution 2 h before surgery. 200 ml of the solution was consumed before surgery in group A and 400 ml of the solution was consumed before surgery in group B. The patients in group B consumed 200 ml of the solution before surgery.

### Perioperative management

#### Preoperative preparation and anesthesia management

All patients took oral laxatives to clean the bowel on the 1st day before surgery, no mechanical enema was performed, and solid food was fasted 6 h before surgery. Two hours before induction of anesthesia, patients in group A consumed a carbohydrate drink containing 5% glucose solution pre-warmed at 37°C, 200 ml and 400 ml in group A and group B. Prophylactic antibiotics were infused intravenously 30 min before surgery, and baseline vital signs (non-invasive blood pressure, electrocardiogram, SpO2, respiratory rate, and body temperature) were monitored. Depth of anesthesia was regulated in real time by Narcotrend monitor (MT MonitorTechnik GmbH & Co.KG, Germany) (maintenance index 37–45). The radial artery was cannulated and linked to a FloTrac/Vigileo system for continuous monitoring of stroke volume (SV) and stroke volume variability (SVV).

The anaesthesia induction protocol comprised the administration of intravenous midazolam (0.05 mg/kg), pentoxyverine hydrochloride (0.4–0.6 mg), and lansoprazole (30 mg), followed by etomidate (0.3 mg/kg), sufentanil (0.4–0.6 µg/kg), and cisatracurium (0.15 mg/kg). The induction was conducted in a rapid manner, and tracheal intubation was performed following preoxygenation with a face mask. Mechanical ventilation parameters were set at FiO2 30%–50%, tidal volume 6–8 ml/kg, and respiratory rate 12–15 breaths/min (I:E = 1:2). Intraoperative maintenance was performed with propofol (3–6 mg/kg/h), remifentanil (0.05–0.2 µg/kg/min), and sevoflurane (1%–1.5% inhalation concentration), with additional inotropic medication if necessary.

#### Intraoperative operations and fluid management

After right subclavian vein cannulation, ultrasound-guided bilateral transversus abdominis plane block (TAP) was performed, with 20 ml of 0.375% ropivacaine injected on each side. Intraoperative fluid management followed a goal-directed strategy (GDFT), with SVV ≤13% as the threshold for volume responsiveness: if SVV >13%, 150 ml of hydroxyethyl starch 130/0.4 sodium chloride injection was rapidly infused, with a 10-minute was completed and reassessed within 10 min. Ustatin (5,000 U/kg) dissolved in crystalloid was infused intravenously to reduce the inflammatory response.

#### Surgical standardization and postoperative management

The surgical operation strictly followed the Japanese Guidelines for the Treatment of Gastric Cancer (4th edition) (12), including the extent of laparoscopic gastric resection, lymph node dissection, and gastrointestinal reconstruction approach. Postoperatively, a nasogastric tube was left in place only when there was a high risk of anastomotic fistula or bleeding risk, and bilateral abdominal drains were routinely placed (the left side was positioned in the splenic fossa, and the right side was located below the left lobe of the liver and adjacent to the gastroesophageal junction). Postoperative analgesia was provided by epidural block combined with non-steroidal anti-inflammatory drugs, and patients were encouraged to get out of bed and wear gradient compression stockings at an early stage. Parenteral nutrition was initiated from postoperative day 1, and the time to return to a liquid diet was determined by the surgeon's assessment. Discharge criteria included normal temperature, voluntary mobility, recovery of gastrointestinal function (at least one bowel movement), oral diet tolerated, manageable pain (oral analgesia) and voluntary discharge of the patient.

#### Outcomes

Primary endpoint: preoperative gastric residual volume: after induction of anesthesia and parallel endotracheal intubation, the gastric contents were completely aspirated under direct vision via an Olympus CV290 gastroscope (Olympus, Japan), connected to a sterile closed collector. Subsequently, the total volume of gastric residual fluid was measured in millilitres using a graduated cylinder.

Secondary endpoints: intragastric acidity levels, preoperative thirst and hunger scores, perioperative blood glucose, electrolyte fluctuations, intraoperative indices and hemodynamic changes: stroke volume (SV) and stroke volume variability (SVV), heart rate, and blood pressure. Immediate operative results, recovery of bowel function, incidence of perioperative complications, postoperative hospitalization and 30-day postoperative readmission.

Intragastric acidity levels: A Delta 320 pH detector (Mettler Toledo, Switzerland) was used to determine the pH of the gastric fluid.

Preoperative thirst and hunger scores: Immediately before induction of anesthesia, patients rated themselves on a 10 cm visual analog scale (VAS) (0 cm = no discomfort, 10 cm = extreme discomfort), which was recorded by an independent research assistant.

Postoperative fluctuations of blood glucose and electrolytes: using the blood glucose value without drinking carbohydrate drinks containing 5% glucose solution before surgery as the baseline, blood glucose levels were monitored dynamically at 4 h, 8 h and 12 h after surgery, and the patients' electrolyte indexes were rechecked at 1 day before surgery and 1 and 3 days after surgery. The degree of fluctuation of electrolytes and blood glucose was calculated to determine the changes in patients' metabolic levels.

Intraoperative indices and hemodynamic changes: Surgical time (minutes), intraoperative bleeding (ml), total rehydration volume (ml), and urine volume (ml) were recorded. Immediately after induction of anesthesia, beat volume (SV) and beat volume variability (SVV), heart rate, and blood pressure were recorded. After oral administration was recorded as T1, after the establishment of anesthesia as T2, and at the beginning of surgery for 1 h as T3, respectively. In the time period of T3, because the anesthesiologist individually adjusted the amount of fluids in the patient during the operation to stabilize the patient's intraoperative hemodynamics, only the first two time points were recorded as T1, T2, and T3. Therefore, only the first two time points were statistically analyzed.

To record postoperative recovery indicators: record time to first postoperative defecation (days); complications and hospitalization outcomes: perioperative complications (e.g., infections, anastomotic fistulas, bleeding, etc.), postoperative hospitalization (days), and 30-day postoperative readmission rates were counted. Operative sequelae were characterized as any treatment-associated morbidity developing during the initial postoperative month. Unplanned hospital readmissions and procedure-related fatalities specifically referred to healthcare facility reentries and lethal outcomes attributable to intervention-related sequelae within the same timeframe.

#### Statistical analysis

Frequency distributions (%) described qualitative measures, while quantitative parameters were expressed as arithmetic mean with dispersion metrics or median values with interquartile dispersion. Parametric two-sample comparisons (continuous data) and nonparametric rank-sum evaluations were applied to numerical datasets, with chi-square contingency analyses or exact probability computations employed for nominal variables. Longitudinal data modeling framework incorporated mixed-effects regression, assigning experimental allocation as stationary parameters and temporal effects (including time-allocation interactions) as stochastic variables. All analyses were performed using SPSS 25.0 (IBM SPSS Statistical Software, IBM, Armonk, NY, USA).

### Sample size estimation

This trial employs an asymmetric equivalence margin of −10 ml to 20 ml for gastric residual volume (GRV) differences, prespecified based on:Clinical evidence: Previous trials demonstrate that GRV reductions ≥10 ml improve postoperative ileus resolution, while increases ≤20 ml do not elevate aspiration risk (7, 11, 13). Biological plausibility: Given preoperative carbohydrate loads (200–400 ml) (14), a 20 ml difference represents a 5%–10% change in volume, which falls within metabolically adaptable thresholds. The standard deviation (SD) of GRV was set at 12.0 ml, derived from historical studies and pilot data (7, 11, 13). With 90% power to detect equivalence (*α* = 0.05), 66 participants per group are required. This calculation allows for a 10% rate of discontinuation and loss to follow-up.

## Results

### Patient characteristics

**Figure 1:**
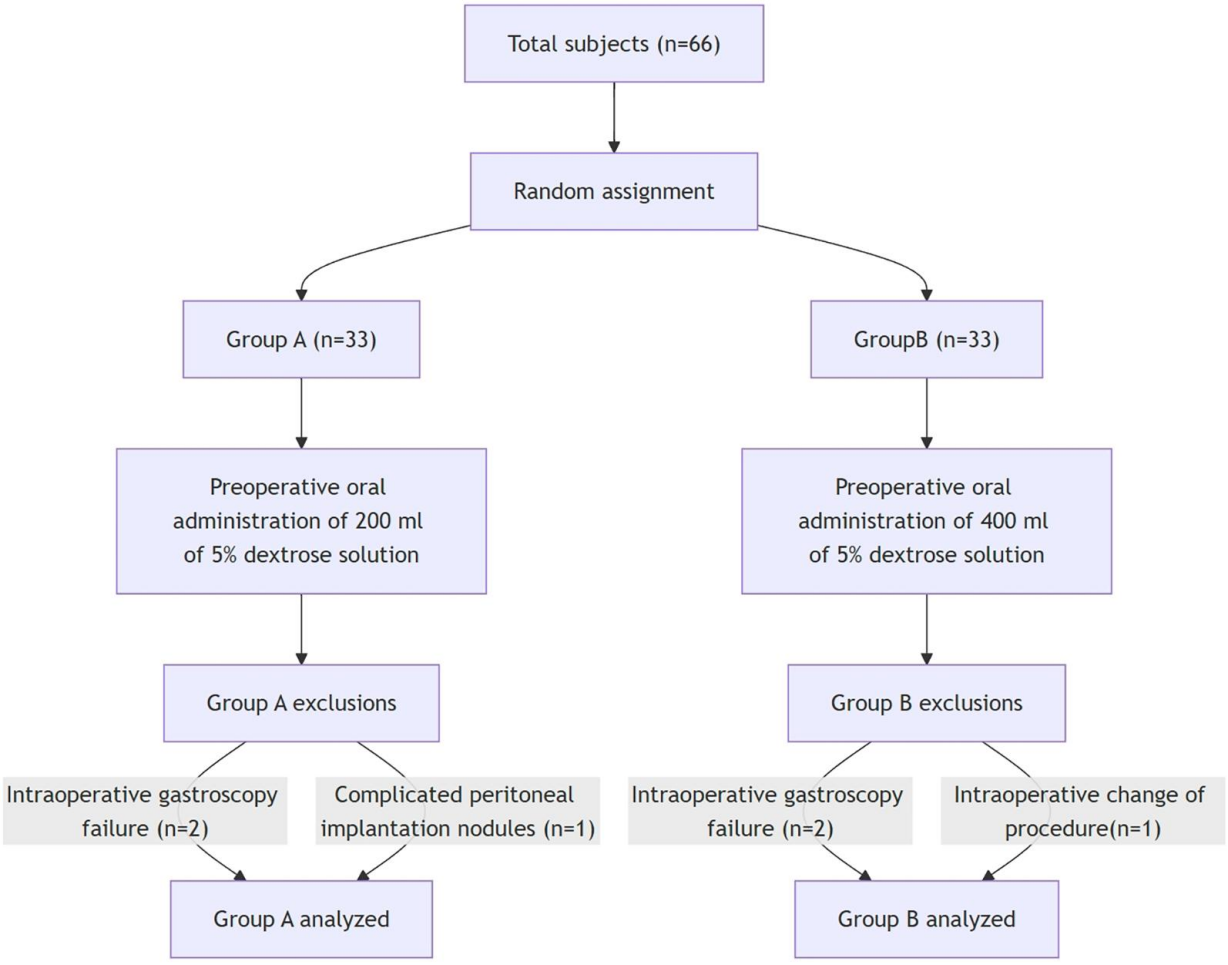
Research flowchart.

The flowchart of the trial is shown in Figure 1. Between April 2021 and December 2024, 66 patients were recruited and randomized into 200 ml group (33 patients) and 400 ml group (33 patients). Two patients in group A were excluded due to failure of intraoperative gastroscopy and one intraoperative finding of peritoneal implantation nodule. Two patients in group B were excluded from group A due to failure of intraoperative gastroscopy and one case of intraoperative change of operative modality by conversion from laparoscopic to invasive surgery (1 case) Finally, 30 patients in group A and 30 patients in group B completed the study.

Baseline characteristics were well balanced between groups A and B. There were no statistically significant differences between groups A and B in terms of age, gender, BMI, tumor location, tumor stage, type of surgery, resection and reconstruction (Table 1).

**Table 1:** Demographic, physiological, and surgical characteristics of patients at baseline.

Variables	A group *n* = 30	B group *n* = 30	*P* value
Age, years, mean ± SD	69.4 ± 6.6	71.2 ± 6.2	0.26
BMI, kg/m^2^, mean ± SD	23.43 ± 3.25	23.44 ± 3.14	0.99
Male	20 (66.7%)	21 (70%)	0.78
Tumor location			0.44
Cardia	12 (40.0%)	15 (50.0%)	
Gastric Corpus	10 (33.3%)	9 (30.0%)	
Gastric Antrum	8 (26.6%)	6 (60.0%)	
TNM classification			0.76
I	10 (33.3%)	8 (26.6%)	
II	6 (60.0%)	7 (23.3%)	
III	14 (46.6%)	15 (50%)	
Type of resection			0.94
Proximal gastrectomy	10 (33.3%)	11 (36.6%)	
Total gastrectomy	13 (43.3%)	13 (43.3%)	
Distal gastrectom	7 (23.3%)	6 (60.0%)	
Type of reconstruction			0.94
Billroth I	10 (33.3%)	11 (36.6%)	
Roux-en-Y	13 (43.3%)	13 (43.3%)	
Billroth II	7(23.3%)	6(60.0%)	

BMI, body mass index.

### Gastric residual volume and pH, and preoperative discomfort

Comparison between the groups showed that the differences in the functional indicators of gastric emptying between the two groups were not statistically significant: gastric juice pH (Group A: 2.57 ± 0.49 vs. Group B: 2.62 ± 0.53, R = 0.14, *P* = 0.67) and gastric residual volume (Group A: 36.4 ± 9.6 ml vs. Group B: 37.7 ± 8.8 ml, R = 0.14, *P* = 0.565) did not present a significant differences. The 95% confidence interval for the difference in gastric residual volume (GRV) between the two groups was −2.85 ml to 6.31 ml, which is entirely within the predefined equivalence interval (−10 ml to +20 ml), meeting the criteria for statistical equivalence. In terms of preoperative subjective feelings, the thirst score (2.03 ± 1.15 vs. 1.57 ± 1.01, R = 0.145, *P* = 0.049) and hunger score (3.1 ± 1.3 vs. 2.3 ± 1.1, R = 0.4, *P* = 0.002) of patients in group B were significantly lower than those in group A. In group B, the thirst score was significantly lower than that of patients in group A. See Table 2 for details.

**Table 2:** Volume and pH of gastric residual and preoperative discomfort.

Variables	A group *n* = 30	B group *n* = 30	Effect size(d)	*P*-value
Gastric residual pH	2.57 ± 0.49	2.62 ± 0.53	0.13	0.886
Gastric residual volume, ml	36.4 ± 9.6	37.7 ± 8.8	0.14	0.565
Preoperative discomfort				
Thirst	2.03 ± 1.15	1.57 ± 1.01	0.145	0.049
Hunger	3.1 ± 1.3	2.3 ± 1.1	0.4	0.002

Cohen's *d* was used to calculate effect sizes for between-group differences in thirst and hunger scores.

### Intraoperative hemodynamic changes

As shown in Table 3, all hemodynamic parameters and heart rate indexes did not present statistical differences between groups A and B. Specifically, the intergroup differences in SVV (12.69 ± 3.21 vs. 11.85 ± 2.56, *P* = 0.105) T1 heart rate (67.6 ± 11.6 vs. 70.6 ± 13.2, *P* = 0.332) and T2 heart rate (68.8 ± 12.87 vs. 67.43 ± 15.90, *P* = 0.693) between the two groups were not significant. In terms of blood pressure indices, intergroup comparisons of mean arterial pressure [MAP (T1):101.3 ± 11.3 vs. 101.7 ± 13.9, *P* = 0.795], and mean arterial pressure [MAP (T2):90.17 ± 13.55 vs. 91.94 ± 12.56, *P* = 0.592] also did not reach statistical significance. In addition, basal cardiac output (C0:4.06 ± 1.09 vs. 3.93 ± 0.98, *P* = 0.700) and cardiac index (CI:2.42 ± 0.62 vs. 2.56 ± 0.76, *P* = 0.601), the cardiac output-related parameters, also did not show significant differences between the two groups.

**Table 3:** Intraoperative hemodynamic blood changes.

Variables	A group *n* = 30	B group *n* = 30	*p*-value
SV (ml)	61.12 ± 4.45	60.57 ± 4.24	0.623
SVV (%)	12.69 ± 3.21	11.85 ± 2.56	0.105
C0(L/min)	4.06 ± 1.09	3.93 ± 0.98	0.700
CI [L/(min/m^2^)]	2.42 ± 0.62	2.56 ± 0.76	0.601
HR (T1) (time/min), mean ± SD	67.6 ± 11.6	70.60 ± 13.2	0.332
HR (T2) (time/min), mean ± SD	68.8 ± 12.87	67.43 ± 15.90	0.693
MAP(T1), (mmHg), mean ± SD	101.30 ± 11.30	101.70 ± 13.90	0.795
MAP(T2), (mmHg), mean ± SD	90.17 ± 13.55	91.94 ± 12.56	0.592

### Results of blood glucose and electrolyte changes

The differences in blood potassium, sodium and calcium levels between the two groups were not statistically significant at all time points. Linear mixed model analysis showed that: (1) for the time effect, the three electrolyte levels showed weak fluctuations postoperatively (F value not reported, *P* > 0.05), and its potential trend needs to be verified by enlarging the sample or prolonging the observation period; (2) the main effect of grouping was not statistically significant (F value not reported, *P* > 0.05); and (3) the interaction between time and grouping was not significant (*P* > 0.05), suggesting that the different rehydration strategies had no significant effect on the electrolyte trends had no significant effect.

In blood glucose analysis, blood glucose levels were significantly lower in group A than in group B at 4 h postoperatively (10.1 ± 2.22 vs. 11.03 ± 3.51 mmol/L, *P* = 0.048, r = 0.26), and at the remaining time points (Glu1: 7.07 ± 2.14 vs. 7.28 ± 2.05; Glu3: 9.54 ± 3.03 vs. 9.33 ± 2.92; Glu4: 8.32 ± 2.84 vs. 8.18 ± 3.05 mmol/L, *P* > 0.05) and outlier-corrected Glu2 (*P* = 0.201) did not differ between groups. Linear mixed models confirmed that (1) there was a significant time effect for blood glucose (F = 33.323, *P* < 0.001) and (2) the group main effect (F = 1.232, *P* = 0.271) and time-group interaction (F = 1.158, *P* = 0.327) were not significant, suggesting that the trend of glucose change was not affected by group factors. The above data are detailed in Table 4.

**Table 4:** Electrochemical and blood sugar level changes.

Variables	A group *n* = 30	B group *n* = 30	*P*-value
Potassium, mmol/L, mean ± SD			
Baseline	4.06 ± 0.32	4.03 ± 0.35	0.719
POD1	4.09 ± 0.314	3.98 ± 0.35	0.250
POD3	4.07 ± 0.35	4.05 ± 0.39	0.789
Sodium, mmol/L, mean ± SD			
Baseline	137.60 ± 2.30	138.40 ± 3.40	0.472
POD1	138.41 ± 1.24	138.94 ± 2.33	0.276
POD3	138.90 ± 2.80	138.60 ± 3.30	0.851
Calcium, mmol/L, mean ± SD			
Baseline	2.16 ± 0.15	2.15 ± 0.16	0.810
POD1	2.25 ± 0.15	2.19 ± 0.17	0.087
POD3	2.14 ± 0.13	2.15 ± 0.14	0.770
Plasma glucose, mmol/L, median (IQR)			
Baseline	6.75 (5.98–7.50)	7.76 (6.20–8.90)	0.796
4 h PO	10.05 (8.46–11.32)	10.90 (8.52–12.32)	0.048*
8 h PO	9.15 (6.67–11.55)	9.55 (8.02–11.8)	0.350
12 h PO	8.25 (6.00–9.62)	8.75 (6.67–10.62)	0.680

Baseline, the day before surgery; IQR, interquartile range; POD, postoperative day; SD, standard deviation; h PO, postoperative hour.

### Perioperative treatment efficacy and convalescence progression

Group A and group B showed a significant difference in operative time (338.3 ± 43.2 vs. 336.7 ± 16.9 min, *P* = 0.447), intraoperative bleeding (95.7 ± 90.5 vs. 104.7 ± 90.8 ml, *P* = 0.263), urinary output (364.7 ± 145.3 vs. 374.3 ± 123.7 ml, *P* = 0.580), total rehydration fluid (2,526.7 ± 477.5 vs. 2,523.3 ± 298.1 ml, *P* = 0.890), time to initial defecation [4(3.25–5.25) days vs. 5(3.75–5.75) days, *P* = 0.470] Differences in length of hospitalization [17(14.0–21.0) days vs. 16(15.0–19.0) days, *P* = 0.530] were not statistically significant (Table 5).

**Table 5:** Early surgical outcomes and postoperative course of patients.

Variables	A group *n* = 30	B group *n* = 30	*P*-value
Operation time, min, median (IQR)	333.5 (331.1–347.7)	333.5 (333.5–337.2)	0.447
Intraoperative blood loss, ml, median (IQR)	65.0 (50.0–100.0)	100.0 (50.0–100.0)	0.263
Intraoperative urine output, ml, mean ± SD	364.7 ± 145.3	374.3 ± 123.7	0.580
Intraoperative fluid infusion, ml, mean ± SD	2,526.7 ± 477.5	2,523.3 ± 298.1	0.890
Time to first flatus, days, median (IQR)	4.0 (3.2–5.2)	5.0 (3.7–5.7)	0.470
Total length of hospitalization, days, median (IQR)	17.0 (14.0–21.0)	16.0 (15.0–19.0)	0.530

### Postoperative complications

The incidence of postoperative complications in Groups A and B was 13.3% (4/30 cases) and 16.7% (5/30 cases), respectively, and the difference between the groups was not statistically significant (*P* = 0.733), with some patients combining multiple complications (Table 5). The distribution of specific complications was as follows: group A: 2 cases of abdominal infection (6.7%), 1 case of intra-abdominal hemorrhage (3.3%), 1 case of anastomotic leakage (3.3%), 1 case of biliary leakage (3.3%), 1 case of gastroparesis (3.3%), 1 case of anastomotic stenosis (3.3%), and 1 case of intestinal obstruction (3.3%); group B: 3 cases of abdominal infection (10.0%), 2 cases of intra-abdominal hemorrhage (6.7%), 1 case (3.3%) of ascites, 1 case (3.3%) of anastomotic leakage, 1 case (3.3%) of duodenal stump leakage, and 2 cases (6.7%) of anastomotic stenosis. Analyzed by the continuity-corrected chi-square test and Fisher's exact test, the differences in all complication types between the two groups were not statistically significant (all *P* > 0.05), suggesting that preoperative oral administration of different amounts of dextrose solution (200 ml vs. 400 ml) did not have a significant effect on the risk of postoperative complications Table 6.

**Table 6:** Early surgical outcomes and postoperative course of patients.

Variables	A group *n* = 30	B group *n* = 30	*P*-value
Total	4 (13.3%)	5 (16.7%)	0.733^a^
Intra-abdominal infection	2 (6.7%)	3 (10.0%)	0.676^b^
Intra-abdominal hemorrhage	1 (3.3%)	2 (6.7%)	0.607^b^
Anastomotic leakage	1 (3.3%)	1 (3.3%)	1.000^b^
Biliary leakage	1 (3.3%)	0 (0.0%)	0.494^b^
Chylous leakage	0 (0.0%)	1 (3.3%)	0.494^b^
Duodenal stump leakage	0 (0.0%)	1 (3.3%)	0.494^b^
Gastroparesis	1 (3.3%)	0 (0.0%)	0.494^b^
Anastomotic stenosis	1 (3.3%)	2 (6.7%)	0.607^b^
Bowel obstruction	1 (3.3%)	0 (0.0%)	0.494^b^

a Continuity correction.

b Fisher's exact test.

## Discussions

### Main findings

In patients undergoing elective laparoscopic radical gastric cancer surgery, neither 200 ml nor 400 ml of 5% dextrose solution taken orally 2 h before surgery significantly increased the volume of gastric remnant (gastric remnant volume (36.4 ± 9.6 in group A vs. 37.7 ± 8.8 ml in group B, R = 0.14, *P* = 0.565). There were no statistically significant differences between groups in gastric fluid acidity (pH: 2.57 ± 0.49 vs. 2.62 ± 0.53, *P* = 0.67) or incidence of perioperative complications (all *P* > 0.05), and in intraoperative hemodynamic indices (e.g., mean arterial pressure, heart rate, and volume per beat variability) (all *P* > 0.05). Although the amount of gastric remnant was slightly higher in the 400 ml group, it was below the safety threshold (<1.5 ml/kg) in all cases, and there was no case of aspiration. It is worth noting that preoperative thirst and hunger scores were significantly lower in the 400 ml group than in the 200 ml group (thirst: 1.57 ± 1.01 vs. 2.03 ± 1.15, *P* = 0.049; hunger: 1.3 ± 1.1 vs. 3.1 ± 1.3, *P* = 0.002). The difference in hunger scores between the two groups was 1.8 (d = 0.4), which was both statistically and clinically significant, exceeding the minimum clinically important difference and potentially benefiting patients. Regarding thirst, however, the difference in scores between the two groups was smaller (d = 0.145), indicating a smaller effect; therefore, its clinical value may be lower than that of hunger scores. These results suggest that increasing fluid intake may further enhance patient comfort. Within a safe dosage range, these results indicate that preoperative oral administration of 400 ml of 5% glucose solution aligns with the metabolic management goals of the Enhanced Recovery After Surgery (ERAS) programme, effectively optimising patients' perioperative experience.

### Safety and efficacy

A rise has been observed in the number of patients who are undergoing surgery which is categorised as intermediate- to high-risk, there is a growing need for accelerated recovery surgery (ERAS) programs based on evidence-based medicine (15). ERAS studies have demonstrated that optimization of perioperative fluid management significantly improves patient prognosis, and the strategy has evolved from traditional rehydration and restrictive rehydration to individualized goal-directed fluid therapy (GDFT) (16). The safety and improved patient experience of carbohydrate drinks as an important measure of ERAS 2 h before surgery has been supported by several studies and included in the guidelines of the American Society of Anesthesiologists (ASA): it is recommended that adult patients with normal gastrointestinal motility consume an appropriate amount of clear liquids 2 h before surgery (4, 14, 17). However, there is still disagreement on the standard of intake, with patients drinking 250 ml preoperatively in some gastrointestinal surgeries (13). In contrast, the Chinese 2021 expert consensus limits the dose to 300 ml, which is halved for patients undergoing gastrointestinal surgery. Although most guidelines use 400 ml as a safe threshold, a fixed dose may ignore the effect of individual differences on gastric emptying and fluid requirements. Studies have shown that gastric emptying time is significantly prolonged in females, patients with abnormal BMI (>25 or <18), and elderly patients (18), and a uniform 400 ml regimen may lead to a blood volume imbalance: insufficient rehydration may induce organ hypoperfusion and impaired oxygen delivery, while excessive rehydration may exacerbate interstitial edema, inflammatory response, and anastomotic fistula risk (19, 20). Therefore, individualized strategies based on goal-directed fluid therapy (GDFT) have become a research hotspot, and GDFT can optimize tissue oxygen supply and reduce complications by dynamically monitoring hemodynamic indexes (e.g., volume per beat variability, pulse pressure variability, etc.) to achieve precise volume management (20, 21). Studies have confirmed that GDFT not only reduces acute postoperative pain intensity, but also enhances gastrointestinal function indicators such as glycopeptide (GAL) by modulating intestinal barrier damage markers such as plasma D-lactate and diamine oxidase (DAO), which in turn improves patients' prognosis (22, 23). As we move forward, there is a need for ongoing research to refine protocols and identify optimal fluid management strategies that are consistent with the principles of ERAS to ensure that patients receive the best possible care tailored to their individual needs. Continued development of clinical practice aims to improve postoperative recovery trajectories and patient prognosis.

### Gastric residual volume and pH and risk of aspiration

Pulmonary aspiration, as a serious anesthesia-related complication, is often caused by aspiration pneumonia due to reflux of acidic gastric contents into the bronchial tree, which is associated with a high rate of mortality and serious complications (24, 25). While traditional preoperative fasting strategies aim to reduce the risk of aspiration through gastric emptying, recent studies have confirmed that the intake of clear liquids (≤3 h) 2 h prior to surgery in patients undergoing elective surgery (both adults and children) does not increase gastric retention or decrease pH (13, 26), and alleviates metabolic disturbances and stress, and has been incorporated into ERAS guidelines for colorectal surgery (7, 27). However, fluid management in gastric cancer patients is more controversial: although the ERAS Society recommends a similar strategy for gastrectomy (6), elevated residual volume within the digestive tract constitutes a significant surgical challenge during procedural execution, while distinct gastrointestinal motility patterns observed in oncological populations require comprehensive mechanistic exploration. While Enhanced Recovery Protocols incorporating abbreviated preoperative fasting and preprocedural carbohydrate loading demonstrate potential advantages in oncologic surgical populations (9–11). Nevertheless, the corpus of evidence for direct measurement of the quantification of gastric contents remains limited. Furthermore, confounding variables inherent in multimodal perioperative protocols may compromise isolation of specific interventions' therapeutic impacts. In the present study, by aspirating gastric residue under direct intraoperative visualization and measuring pH, we confirmed that gastric cancer patients who consumed a carbohydrate drink containing 5% glucose solution 2 h before surgery did not increase the volume of gastric residue or decrease pH with the change in volume, and the results generally complemented and corroborated with previous studies (28).

In terms of patient preoperative comfort, the 400 ml group further alleviated preoperative mouth (*p* < 0.05, effect size = 0.145) and hunger symptoms (*p* = 0.002, effect size *r* = 0.40) compared to the 200 ml group, which is in line with the results reported in other studies of 250 ml glucose solution to improve the subjective experience of patients (29, 30). In terms of patient preoperative comfort, the 400 ml group further alleviated preoperative mouth (*p* < 0.05, effect size = 0.145) and hunger symptoms (*p* = 0.002, effect size *r* = 0.40) compared to the 200 ml group, which is in line with the results reported in other studies of 250 ml glucose solution to improve the subjective experience of patients (31). Therefore, this study found that increasing the volume of rehydration fluid within the safe range can further optimize patient comfort without increasing the risk of aspiration.

### Glucose fluctuations and electrolyte changes

Previous studies have shown that surgical stress induces enhanced catabolism and hyperglycemic state, whereas traditional preoperative midnight fasting may further exacerbate insulin resistance, aggravate patient discomfort and reduce intravascular volume, especially in patients with mechanical bowel preparation (32, 33). Preoperative oral carbohydrate (CHO) loading, on the other hand, has been shown to mitigate these effects by improving metabolic homeostasis (34). Focusing on non-diabetic gastric cancer surgical patients, the results of this study showed that the difference in blood glucose between the two groups was not statistically significant (*p* > 0.05) except at 4 h postoperatively (T2 time point).T2 time point analysis showed that blood glucose levels were significantly higher in the 400 ml group than in the 200 ml group (*p* = 0.048) but after further exclusion of the abnormally high values in the 400 ml group (20.8, 18.9, 17.9 mmol/L) the difference disappeared (*p* = 0.201). It is worth noting that patients with hyperglycaemia, characterised by abnormally elevated blood glucose levels, typically have higher BMI values. Analysis of BMI groups revealed that in patients with higher body weight, the 400 ml dose may increase the risk of hyperglycaemia (a difference of 42.85 percentage points in incidence rate), but the sample size was small and did not reach statistical significance (*p* = 0.267). In Group B (400 ml), the risk of hyperglycaemia was significantly higher in overweight patients than in underweight patients (*p* < 0.001), indicating that BMI is a potential risk factor for hyperglycaemia, particularly at higher carbohydrate doses. The reason that the difference in blood glucose between the two groups was not statistically significant for the rest of the overall time points may be that we used a 5% glucose solution rather than the carbohydrate-rich (12.5%) solution typically used in other studies; thus, less glucose was ingested and therefore had a lower impact on metabolism and stress response. The present study confirms the safety of preoperative supplementation with a low-dose CHO solution (5%) in nondiabetic gastric cancer patients without increased metabolic risk. Implications for clinical practice are as follows: (1) For elderly or obese patients (BMI > 25 kg/m^2^), individualized adjustment of fluid supplementation (e.g., a reduction of 50–100 ml) and enhanced intraoperative glucose monitoring are recommended; (2) preoperative assessment needs to integrate gastric emptying function and metabolic markers to optimize fluid management strategies under the ERAS pathway. Future dose-response studies are needed to clarify the impact of obesity-related metabolic defects on the safety of fluid replacement.

### Intraoperative hemodynamic changes

GDFT emphasizes precise volume management through dynamic monitoring of hemodynamic indices (e.g., volume per beat variability, cardiac output) covering the entire preoperative, intraoperative, and postoperative phases to reduce the risk of organ hypoperfusion and fluid overload (35). Preoperative oral carbohydrate (CHO) fluids, as one of the core measures of ERAS, may play a protective role by maintaining metabolic homeostasis, attenuating stress response and improving cardiac function (36). In this study, we focused on the effects of preoperative fluid replacement differences on patients and monitored hemodynamic indices (e.g., mean arterial pressure, heart rate) from the time of anesthesia-induced intubation to 1 h after surgery. The results showed that there was no significant difference in cardiac load-related indices between the 400 ml group And the 200 ml group (*p* > 0.05), confirming the safety of preoperative oral CHO fluids. However, subgroup Analysis suggested that patients with BMI > 25 kg/m^2^ may have inadequate fluid replenishment (15% increased incidence of SVV > 13%), which can be effectively compensated by intraoperative GDFT adjustment (e.g., replenishment increased by 200–300 ml). Stratified analysis by BMI showed that 400 ml may be more optimal for patients with high body weight (14.3% reduction in the incidence of fluid deficiency), but the sample size was too small to be statistically significant. The above findings suggest that a standardized preoperative rehydration regimen (e.g., 400 ml) is safe and feasible in the majority of patients, but individualized modification in conjunction with intraoperative ambulatory monitoring is needed for those with obesity or metabolic abnormalities. Future studies should further explore preoperative rehydration algorithms based on parameters such as BMI and gastric emptying rate to improve fluid management strategies under the ERAS pathway.

### AI perspectives in future ERAS studies of preoperative water intake

The integration of artificial intelligence (AI) in preoperative rehydration management offers the potential to revolutionize individualized care under accelerated recovery surgery (ERAS) pathways. Current ERAS guidelines, while recommending standardized rehydration protocols, do not adequately incorporate heterogeneous patient characteristics (e.g., age, gender, BMI, gastric motility, and surgical stress). AI predictive models constructed based on large-scale language models (LLMs) and machine learning algorithms can dynamically integrate the aforementioned multidimensional data to generate individualized rehydration recommendations. For example, by monitoring the preoperative gastric emptying rate in real time via wearable devices or imaging biomarkers, combined with metabolic characteristics and demographic parameters, AI can accurately predict the optimal rehydration volume to ensure intravascular volume while minimizing the risk of aspiration. In addition, the reinforcement learning framework can dynamically optimize the rehydration strategy based on intraoperative hemodynamic indicators (e.g., beat-to-beat variability) and postoperative recovery data (e.g., electrolyte balance), which is in line with the principles of goal-directed fluid therapy (GDFT) (20).

### Limitations

Our study has several limitations. First, this study was a single-center, small sample size (*n* = 60) exploratory trial, which may affect statistical efficacy and extrapolation of results. Future multicenter, large-sample studies with artificial intelligence and wearable devices (e.g., continuous glucose monitors) are needed to dynamically optimize rehydration dosage and timing, as well as to assess postoperative long-term outcomes (e.g., complication rates, quality of survival) Second, we excluded diabetic patients from the trial due to safety considerations, and carbohydrate metabolism abnormalities in such populations may have a specific need for a preoperative rehydration strategy, which needs to be follow-up targeted studies (3). Due to limitations imposed by domestically available products and considerations regarding surgical safety, this study utilized a 5% glucose solution, precluding comparison with higher-concentration carbohydrate beverages (e.g., 12.5% concentration) or composite nutrients (e.g., whey protein), which have been demonstrated to improve nitrogen balance and muscle metabolism (11, 37–39). Although the study design strictly followed the ERAS guidelines and the metabolic characteristics of the Chinese population, the results still have limitations in terms of external validity and generalisability due to considerations of population similarity and metabolic differences, as well as regional variations in the ERAS guidelines. In the future, stepped concentration trials could be designed to investigate the dose-effect relationship of different formulations on gastric emptying, blood glucose fluctuations, and postoperative recovery. Although we would like to explore the differences in conventional carbohydrate loading for individualization, due to the sample size and experimental results for the optimal solution or regimen (40). We can only give relevant notes on the direction and trend, and have not been able to formulate them. Future studies should reasonably extrapolate hypotheses and design a stepwise validation plan (first multi-centre validation in Asia, followed by adaptive trials in Europe and the United States) to gradually establish a perioperative nutritional management framework for gastrectomy that is applicable across populations.

## Conclusions

In gastric cancer patients undergoing elective laparoscopic radical gastric cancer surgery, consumption of 200 ml compared with 400 ml of carbohydrate beverage containing 5% dextrose solution 2 h before surgery did not significantly increase gastric residual volume, acidity, or perioperative complications, and no significant differences were seen in intraoperative hemodynamics. Preoperative consumption of 400 ml of carbohydrate beverage containing 5% glucose solution further reduced thirst and hunger scores compared to the 200 ml group. Individualized adjustment of fluid replacement is recommended for elderly or obese patients.

## Data Availability

The original contributions presented in the study are included in the article/Supplementary Material, further inquiries can be directed to the corresponding author.
